# Real-time analysis of the binding of fluorescent VEGF_165_a to VEGFR2 in living cells: Effect of receptor tyrosine kinase inhibitors and fate of internalized agonist-receptor complexes

**DOI:** 10.1016/j.bcp.2017.04.006

**Published:** 2017-07-15

**Authors:** Laura E. Kilpatrick, Rachel Friedman-Ohana, Diana C. Alcobia, Kristin Riching, Chloe J. Peach, Amanda J. Wheal, Stephen J. Briddon, Matthew B. Robers, Kris Zimmerman, Thomas Machleidt, Keith V. Wood, Jeanette Woolard, Stephen J. Hill

**Affiliations:** a Cell Signalling and Pharmacology Research Group, School of Life Sciences, University of Nottingham, Nottingham, United Kingdom; b Promega Corporation, Madison, WI, USA

**Keywords:** VEGF, VEGFR2, BRET, Ligand binding, Receptor tyrosine kinase inhibitors

## Abstract

Vascular endothelial growth factor (VEGF) is an important mediator of angiogenesis. Here we have used a novel stoichiometric protein-labeling method to generate a fluorescent variant of VEGF (VEGF_165_a-TMR) labeled on a single cysteine within each protomer of the antiparallel VEGF homodimer. VEGF_165_a-TMR has then been used in conjunction with full length VEGFR2, tagged with the bioluminescent protein NanoLuc, to undertake a real time quantitative evaluation of VEGFR2 binding characteristics in living cells using bioluminescence resonance energy transfer (BRET). This provided quantitative information on VEGF-VEGFR2 interactions. At longer incubation times, VEGFR2 is internalized by VEGF_165_a-TMR into intracellular endosomes. This internalization can be prevented by the receptor tyrosine kinase inhibitors (RTKIs) cediranib, sorafenib, pazopanib or vandetanib. In the absence of RTKIs, the BRET signal is decreased over time as a consequence of the dissociation of agonist from the receptor in intracellular endosomes and recycling of VEGFR2 back to the plasma membrane.

## Graphical abstract

**Figure:**
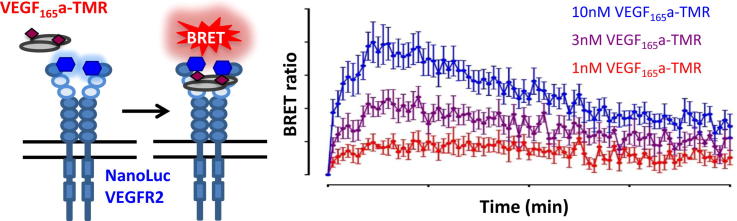

## 1 Introduction

Vascular endothelial growth factor A (VEGF-A) is an important mediator of tumour-induced angiogenesis [1], [2], [3], [4]. Multiple isoforms of VEGF-A exist as a consequence of alternative exon splicing [1], [5], [6]. Furthermore, alternative splicing in exon 8 of the VEGF-A gene can generate additional isoforms with different pharmacological properties [5], [6]. For example, VEGF_165_b has been reported to be a partial agonist at VEGFR2 [7], [8], [9], [10] and an inhibitor of angiogenesis *in vivo* [5].

VEGF family members bind to three VEGF receptors (VEGFR1, VEGFR2 and VEGFR3) with differing selectivity profiles [1], [2]. VEGFR2 is the major regulator of VEGF-driven responses in vascular endothelial cells including permeability, proliferation, invasion, migration and angiogenesis [1], [2]. The binding of VEGF to VEGFRs leads to a change in conformation of their intracellular domains and auto- or trans-phosphorylation of specific tyrosine residues of the receptor dimer resulting in the activation of different intracellular signalling cascades [11], [12], [13], [14]. These signalling pathways are relatively well understood with tyrosine residues Y1175 and Y1214 in the human VEGFR2 being two important auto-phosphorylation sites activated by VEGF binding and tyrosine kinase activation. Phosphorylation of these specific residues creates binding sites for key intracellular signalling proteins such as Grb2, PLCγ and SHC [14], [15], [16].

Receptor tyrosine kinases (RTKs) can exist either as preformed dimers (e.g. the insulin receptor) or dimerize upon ligand binding [16], [17], [18]. Detailed biophysical studies with purified recombinant receptors and receptor domains have provided some insights into the requirement of ligand binding for the formation of VEGFR2 receptor dimers [11], [12]. Ligand binding to VEGFR2 requires Ig-like domains D2 and D3 in the extracellular portion of the receptor [11], [19], [20]. Structural analysis [21], [22] and X-ray crystallography [23], [24], [25] has suggested that ligand binding to VEGFR-2 promotes receptor dimerization. Detailed analysis of the interaction of VEGF with purified recombinant D2-D3 domains of VEGFR2 confirmed that ligand/receptor complexes consisted of two receptor molecules and one VEGF ligand dimer. Furthermore, in the absence of ligand, these D2-D3 recombinant domains are monomeric [23].

Although detailed quantitative characterisation of the interactions of RTK ligands with full-length target receptors in living cells is largely lacking, approaches to achieve this have been suggested from research into other cell surface receptor families. For example, significant advances in our knowledge of G protein-coupled receptors (GPCRs) have come from quantitative analysis of ligand-receptor interactions in living cells [26], [27], [28]. Furthermore, the recent development of new fluorescent ligand technologies has provided significant advances in our ability to unravel the complexities of these interactions [27], [29]. We have recently developed a novel and quantitative approach to monitor ligand-receptor interactions in living cells based on bioluminescence resonance energy transfer (BRET) [30]. This approach monitors energy transfer from a new bright luminescent protein (NanoLuc luciferase) genetically fused to the N-terminus of a GPCR to a red or green fluorescent ligand specifically bound to the target receptor.

Here we have developed a method for stoichiometric labeling of VEGF_165_a at a single N-terminal cysteine, which retains its full agonist activity. This tetramethyl-rhodamine (TMR) labeled VEGF (VEGF_165_a-TMR) has been used in conjunction with VEGFR2 genetically fused to NanoLuc to provide detailed quantitative information on ligand binding characteristics, the effect of receptor tyrosine kinase inhibitors and the internalization of agonist-receptor complexes in living cells.

## 2 Materials and methods

### 2.1 Materials

All recombinant human VEGF ligand isoforms were purchased from R&D Systems (Abingdon, UK). Cediranib, pazopanib, sorafenib and vandetanib were supplied by Sequoia Research Products (Pangbourne, UK). ONE-Glo™ luciferase and HaloTag® ligands were purchased from Promega Corporation (Wisconsin, USA).

### 2.2 Construct for producing labeled VEGF_165a_

The VEGF_165_a cDNA was synthetically generated by Gene Dynamics LLC (Oregon, USA) and sub cloned into a modified pFN21 HaloTag CMV Flexi Vector (G2821; Promega Corporation, USA) containing an IL6 secretion signal. The linker sequence separating the two fusion partners was replaced with a linker encoding EPTTEDLYFQCDN.

### 2.3 Constructs for VEGFR2

VEGFR2 was subcloned from a plasmid obtained from Origene (NM_002253; Maryland, USA). For N terminal NanoLuc tagged constructs, VEGFR2 was cloned into a pF-sNnK CMV/neo vector (N1321; Promega Corporation, USA) encoding a fusion of the signal peptide sequence of IL-6 onto the N terminus of NanoLuc (NLuc). This resulted in open reading frames (ORFs) which encoded NLuc fused via a Gly-Ser-Ser-Gly(AIA) linker to the N terminus of VEGFR2 (termed NLuc VEGFR2). For N terminal HaloTag constructs, VEGFR2 cDNA was cloned into a pFN21A CMV/neo flexi vector encoding a fusion of the signal peptide sequence of IL-6 onto the N terminus of HaloTag. The resultant ORFs encoded HaloTag fused to the N terminus of VEGFR2 via a EPTTEDLYFQSDN(AIA) linker (Halo VEGFR2). C terminal HaloTag VEGFR2 was created by cloning VEGFR2 cDNA into a pFC27K CMV/neo flexi vector (G8431; Promega Corporation, USA). This fused HaloTag onto the C terminus of VEGFR2 via a (VSL)EPTTEDLYFQSDND linker, resulting in VEGFR2-HaloTag (VEGFR2-Halo).

### 2.4 Synthesis and purification of VEGF_165_a-TMR

VEGF_165_a was transiently expressed as a secreted HaloTag fusion protein in 1 L of HEK293T cells and purified using the HaloTag mammalian protein detection and purification system (G6795; Promega Corporation, USA) following the manufacturer’s recommendations. Briefly, after 72 h of expression, the media containing the secreted fusion protein was collected and applied onto 1.8 ml of pre-equilibrated HaloLink beads. Binding to the beads was conducted at 4 °C over night (16–18 h) with constant end over end rotation. Following three washes of the beads, each for 10 min, VEGF_165_a was released from the beads by proteolytic cleavage using 700 units of HaloTEV in the presence of 100 µM TCEP (Sigma-Aldrich, USA). For the generation of a labeled VEGF_165_a, the HaloTEV proteolytic release was done in the presence of 100 µM TCEP and 4× molar excess of 6-TMR-PEG-CBT [31] over the expressed fusion protein. To determine the amount of 6-TMR-PEG-CBT (moles) required for the proteolytic/labeling reaction, the amount of expressed HaloTag:VEGF_165_a (moles) was calculated as described previously [33]. The proteolytic/labeling reaction was conducted at 4 °C over night (16–18 h) with constant mixing. This step generated VEGF_165_a with an N-terminal cysteine that served as single point of conjugation with 6-TMR-PEG-CBT. The purified labeled VEGF_165_a was dialyzed for 24 h (50 mM HEPES, 150 mM NaCl; Sigma-Aldrich, USA) to remove the unconjugated 6-TMR-PEG-CBT and TCEP. The yield from 1L commonly ranged between 0.5 and 1 mg. The purified protein was stored without any further concentration in the presence of 2.5 mg/ml bovine serum albumin (BSA; Millipore, USA; final concentration) in aliquots at -80 °C (final concentration ranged between 80 and 300 µg/ml).

### 2.5 Cell culture

All HEK293T cell lines were grown in Dulbecco’s Modified Eagle’s Medium (DMEM 6429; Sigma-Aldrich, UK) supplemented with 10% fetal calf serum (Sigma-Aldrich, UK) at 37 °C/5% CO_2_. All stable and transient transfections were performed using FuGENE HD (Promega Corporation, USA) according to the manufacturer’s instructions at a reagent to cDNA ratio of 3:1.

### 2.6 Stable cell line generation and maintenance

Stable HEK293T NanoLuc VEGFR2 (NLuc-VEGFR2) and HEK293T VEGFR2-HaloTag cells were cultured in Dulbecco’s modified Eagle’s medium (DMEM; D6429; Sigma-Aldrich, UK) supplemented with 10% fetal calf serum and incubated at 37 °C/5% CO_2_. Cell passaging was performed when cells reached 80% confluency using PBS (Lonza, Switzerland) and trypsin (0.25% w/v in versene; Lonza, Switzerland). We confirmed that these cell lines were mycoplasma free.

### 2.7 Dimerization and glycosylation assays

Dimerization assay: purified VEGF_165_a-TMR was analysed on SDS-PAGE in the presence and absence of 100 mM dithiothreitol (DTT; Sigma-Aldrich, UK) and then scanned on a Typhoon 9400 fluorescent imager (GE Healthcare, USA), (Excitation = 532 nm; Emission = 580 nm).

Glycosylation assay: purified VEGF_165_a-TMR was treated with PNGase (Promega Corporation, USA) as recommended by the manufacturer. Treated and untreated VEGF_165_a-TMR were analysed on SDS-PAGE and then scanned on a Typhoon 9400 fluorescent imager (GE Healthcare, USA), (Excitation = 532 nm; Emission = 580 nm).

### 2.8 Liquid chromatography–tandem mass spectrometry (LC–MS/MS) analysis

VEGF_165_a-TMR and VEGF_165_a purified in the same manner were suspended in 50 mM ammonium bicarbonate (0.1 mg/ml; Sigma-Aldrich, USA) and reduced with 25 mM TCEP at 50 °C for 1 h. Following reduction, the appropriate protease (LysC, GluC, or trypsin/LysC from Promega Corporation, USA) were added to the sample (1:20 enzyme/substrate ratio) and subjected to proteolysis for 3 h at 37 °C with shaking at 400 rpm (Eppendorf Thermomixer). Following this step, the reaction was quenched by the addition of formic acid (1% final concentration; Sigma-Aldrich, USA) and subjected to LC-MS/MS analysis on a Q Exactive Hybrid Quadrupole-Orbitrap Mass Spectrometer (Thermo Fisher Scientific, USA) operating with an MS resolution of 70,000.

Analysis of labeling specificity and efficiency: the mass of the modification (i.e. the mass of the dye after covalent attachment to an N-terminal cysteine) was determined to be 817.28 Da. MS-product component of the Protein Prospector software (University of California, USA) was used to calculate the mono-isotopic masses of the modified and unmodified peptides at different charge states (Z = 2–5 were considered; Table 1). Extracted Ion Chromatograms were generated using Xcalibur (Thermo Fisher Scientific, USA). As equimolar amounts of samples were loaded onto the LC-MS/MS, labeling efficiency could also be determined by comparing the integrated peak areas of the unmodified proteolytic peptides in the labeled and unlabeled samples (Table 1).

**Table 1:** LS-MS/MS profiles for (a) unlabeled and (b) labeled peptide fragments obtained following protease digestion of VEGF_165_a or VEGF_165_a-TMR. In (a) the labeling efficiency of VEGF_165_a-TMR was determined by comparing the integrated peak areas of the unmodified proteolytic peptides in the labeled and unlabeled samples that were digested with LysC, GluC or Trypsin/LysC. The analysis indicated >98% labeling efficiency. (b) Observed mass of the labeled peptides containing the N-terminal cysteine confirmed a mass increase of 817 Da due to covalent attachment of the 6-TMR-PEG-CBT. ^*^Mono-isotopic mass at a single charge assuming the peptide takes up a single proton. Mono-isotopic mass [M+H] is calculated as (*m*/*z*) * z − (z − 1).

*(a) Unlabeled peptides*									
Peptide Sequence	Mass/charge (*m*/*z*)	Charge (z)	Mass [M+H]^*^	Retention Time (min)	Protease	Peak Area	Protease sample	%	% labeled
CDNAPM(Ox)AEGGGQNHHEVVK	670.29	3	2008.86	8.06	Trypsin/LysC	1.04E+06	labeled	0.1	99.9
CDNAPM(Ox)AEGGGQNHHEVVK	670.29	3	2008.86	8.29	Trypsin/LysC	1.20E+09	unlabeled	100	
CDNAPMAEGGGQNHHEVVK	664.96	3	1992.87	10.45	LysC	1.17E+08	labeled	0.9	99.1
CDNAPMAEGGGQNHHEVVK	664.96	3	1992.87	10.8	LysC	1.25E+10	unlabeled	100	
CDNAPM(Ox)AE	425.65	2	850.31	14.29	GluC	1.88E+07	labeled	1.3	98.7
CDNAPM(Ox)AE	425.65	2	850.31	13.97	GluC	1.42E+09	unlabeled	100	

*(b) Labeled peptides*							
Peptide Sequence	Mass/Charge (*m*/*z*)	Charge (z)	Mass of labeled peptide [M+H]^*^ (A)	Mass of un-labeled peptide [M+H]^*^ (B)	Mass difference (A–B)	Retention Time (min) (labeled)	Protease
CDNAPM(Ox)AEGGGQNHHEVVK	707.29	4	2826.14	2008.86	817.28	23	Trypsin/LysC
CDNAPMAEGGGQNHHEVVK	937.38	3	2810.14	1992.87	817.27	24.4	LysC
CDNAPM(Ox)AE	556.53	3	1667.59	850.31	817.28	35.3	GluC

### 2.9 Fluorescence correlation spectroscopy (FCS)

Solution FCS was performed essentially as described previously [32]. Briefly, FCS was performed in Nunc Labteck 8 well chambered coverglasses (ThermoFisher Scientific, UK) using a LSM510 NLO Confocor3 microscope equipped with a c-Apochromat 40×/1.2NA water-immersion objective (Zeiss, Germany). The confocal volume was placed in the solution 200 μm above the surface of the coverslip. Beam paths were calibrated using 20 nM Rhodamine 6G (R6G; *D* *=* 2.8 × 10^−10^ m^2^/s; Sigma-Aldrich, UK) prepared in high performance liquid chromatography grade water (Chromasolv®; Sigma-Aldrich, UK) using both 488 nm and 561 nm laser lines. Calibration measurements were collected using 10 × 10 s reads. FCS measurements were performed using a range of VEGF_165_a-TMR concentrations (0.25–10 nM) in Hanks Buffered Saline Solution (HBSS) with or without 0.1% protease-free BSA (A7030; Sigma-Aldrich, UK). Additional measurements were recorded in reducing conditions consisting of HBSS/0.1% protease free BSA supplemented with 10 mM DTT, pre-incubated for 30 min at 37 °C. Fluctuation measurements were performed using 561 nm excitation light, with emission collected through a long-pass LP580 filter. Time-correlated measurements were recorded for up to 120 min with laser power set to 20% (AOTF set to 10; equivalent to 0.39 kW/cm^2^), measuring 2 × 10 s consecutive reads per well. Autocorrelation analysis was performed using Zen2010 software (Zeiss, Germany), with all data fitted to a single one-component, free 3D Brownian diffusion model, including a pre-exponential for triple state of the fluorophore.

### 2.10 NFAT luciferase reporter gene assay

NFAT luciferase reporter gene activity was monitored in a HEK293T ReLuc2P (Promega Corporation, USA) suspension cell line stably expressing VEGFR2 as described previously [10].

### 2.11 NFAT luciferase reporter gene assay in NLuc VEGFR2 cells

HEK293T NLuc VEGFR2 cells were seeded 48 h prior to assay onto poly-D-lysine (0.1 mg·mL^−1^) coated white sided 96 well plates (655089; Greiner Bio-One, UK) at a density of 25,000 cells/well. Medium was removed and replaced with 100 μl serum free DMEM and cells incubated for a further 24 h. On the day of the assay, incubation medium was replaced with serum free DMEM/0.1% protease free BSA (vehicle). Where appropriate, cells were preincubated for 30 min with different concentrations of RTKIs. Ligands were added in 10 μl to quadruplicate wells, and plates incubated for 5 h at 37 °C/5% CO_2_. Assay medium was then removed and replaced with 50 μl vehicle per well. 50 μl of ONE-Glo™ Luciferase assay reagent (E6120; Promega Corporation, USA) was then added to each well and luminescence measured using a TopCount platereader (Perkin Elmer, UK).

### 2.12 NanoBRET ligand binding

HEK293T NLuc-VEGFR2 cells were seeded 24 h prior to assay onto white sided poly-D-lysine coated 96 well plates at a density of 40,000 cells/well. On the day of the assay, medium was replaced with 50 μl per well HBSS/0.1% protease free BSA. Where appropriate, RTKIs were added 30 min prior to agonist. For competition experiments, a fixed concentration of fluorescent VEGF_165_a-TMR was added simultaneously with competing unlabeled VEGF isoforms to triplicate wells for the time specified (10, 30 or 60 min) and plates co-incubated at 37 °C in the dark. For saturation experiments a range of concentrations of VEGF_165_a-TMR were added in the presence or absence of a competing concentration (30 nM) of unlabeled VEGF_165_a and incubated for 60 min at 37 °C in the dark. The NanoLuc substrate furimazine (N1130; Promega Corporation, USA) was then added at a final concentration of 10 μM. In timecourse experiments, VEGF_165_a-TMR (2, 10 or 20 nM) was added to duplicate wells and incubated for 1 to 120 min before 10 μM furimazine was added. For all experiments, sequential recordings were performed at room temperature using a PHERAstar FS plate reader (BMG Labtech, Germany) using 460 nm (80 nm bandpass; donor NanoLuc emission) and >610 nm longpass filters (acceptor emission). Raw BRET ratios were calculated from the ratio of acceptor to donor emission values. For real-time ligand association kinetics experiments, cells were incubated with vehicle for 30 min at 37 °C. Furimazine (10 μM) was then added and plates equilibrated at 37 °C in the PHERAstar FS plate reader for 5 min. The required concentration of VEGF_165_a-TMR was then added to the appropriate wells, and the plate then immediately returned to the PHERAstar and luminescence and fluorescence readings taken every 30 s for 16 min or 120 min at 37 °C.

### 2.13 Measurement of VEGF_165_a-TMR internalization and VEGFR2 activation status using a widefield platereader

HEK293T NLuc-VEGFR2 cells were seeded 24 h prior to assay onto poly-d-lysine coated black sided 96 well plates (655090; Greiner Bio-One, UK) at a density of 30,000 cells/well. On the day of the assay, medium was replaced with 100 μl per well serum free DMEM/0.1% protease free BSA. Cells were incubated with increasing concentrations of VEGF_165_a-TMR in triplicate wells for 60 min. For competition experiments, VEGF_165_a-TMR (3 nM) was added simultaneously with increasing concentrations of unlabeled VEGF_165_a for 60 min_._ At the end of the incubation period, cells were washed using PBS and fixed using 3% paraformaldehyde (Sigma-Aldrich, UK)/PBS for 10 min at room temperature. Nuclei were then stained using bisBenzimide H 33342 trihydrochloride (2 μg/ml in PBS; Sigma-Aldrich, UK). Cells were imaged using a IX MDC Micro widefield platereader (Molecular Devices, USA) fitted with a 20× Plan Apochromat NA0.6 extra long working distance objective, with 4 sites imaged per well using DAPI (H33342; 405 nm excitation, 447/60 emission) and TRITC (VEGF_165_a-TMR; 560 nm excitation, 607/34 nm emission) filter settings. To track the time course of internalization of VEGF_165_a-TMR/VEGFR2 complexes and the corresponding activation status of the VEGFR2 within these cells, 3 nM VEGF_165_a-TMR was added at set time points (reverse time-course, 120–1 min) in serum free DMEM/0.1% BSA. Cells were then fixed using 3% PFA/PBS for 15 min at room temperature, permeabilised using 0.025% Triton-X-100 in PBS (Sigma-Aldrich, UK) and non specific fluorescence quenched using 3% BSA/1% glycine wash for 30 min at room temperature. Non-specific antibody binding was blocked at room temperature using 30 min incubation with 10% chicken serum in PBS, prior to labeling with a rabbit antibody targeting either pY1175 (Cell Signalling No. 2478) or pY1214 (Cell Signalling No. 2477) residues of VEGFR2 (1:100 dilution in 10% chicken serum in PBS; overnight at 4 °C). The next day cells were labeled with a secondary antibody (chicken anti rabbit AlexaFluor-488 (A-21441; ThermoFisher Scientific); 1:500 dilution in 10% chicken serum/PBS; 60 min at room temperature). Cells were then washed twice in PBS, nuclei stained using H33342 (2 mg/ml in PBS; 10 min at room temperature) and imaged using a IX Micro widefield platereader using DAPI (H33342; 405 nm excitation, 447/60 emission), FITC (anti pY1214 antibody; 488 nm excitation, 514 emission) and TRITC (VEGF_165_a-TMR; 560 nm excitation, 607/34 nm emission) filter settings.

To track the time course of VEGFR2-HaloTag internalization, cells were seeded 24 h prior to assay onto poly-d-lysine coated black sided, clear flat bottomed 96 well plates at a density of 30,000 cells/well. On the day of the assay, medium was replaced with 50 μl per well 10% fetal calf serum/DMEM containing 1 μM HaloTag-TMR cell permeant fluorescent ligand (15 min at 37 °C/5% CO_2_; G8251, Promega Corporation, USA). Cells were then washed 3 times with 80 μl 10% fetal calf serum/DMEM, prior to incubation at 37 °C/5% CO_2_ for 30 min. Medium was then removed and replaced with 45 μl per well assay buffer (HBSS/0.1% BSA). Unlabeled 3 nM VEGF_165_a was added at set time points (37 °C). Cells were then washed using PBS, fixed (3% PFA/PBS) and nuclei stained using H33342 (2 mg/ml in PBS; 10 min at room temperature). Cells were imaged using a IX Micro widefield platereader with 4 sites imaged per well using DAPI (H33342; 405 nm excitation, 447/60 emission) and TRITC (HaloTag-TMR; 560 nm excitation, 607/34 nm emission) filter settings.

Data were analysed using a granularity algorithm (MetaXpress 2.0, Molecular Devices, USA) with vesicles subjectively set in terms of size (3–15 μm) and pixel grey levels in respect to negative and positive plate controls. Data were pooled from 3 to 8 independent experiments and expressed as number of vesicles on a per cell basis.

### 2.14 Confocal Zeiss LSM510 imaging

HEK293T NLuc-VEGFR2 cells were seeded 48 h prior to assay onto poly-D-lysine coated 8 well plates (Nunc LabTek, Thermo Fisher Scientific, UK) at a density of 20,000 cells/well. Medium was replaced with serum free DMEM before cells were incubated for a further 24 h at 37 °C/5% CO_2_. On the day of the assay, cells were washed three times with HBSS/0.1% protease free BSA (vehicle). Receptor tyrosine kinase inhibitors were added at this point as aliquots to the appropriate wells and cells incubated for 30 min at 37 °C. Cells were then stimulated with 10 nM VEGF_165_a-TMR for 30 min at 37 °C in the dark. Cells were imaged using a Zeiss LSM510 fitted with a 63× Pan Apochromat oil objective (1.4NA) using Argon 546 laser excitation, a long pass 540 filter and a pinhole diameter of 1 Airy unit. All images were taken at 1024 × 1024 pixels per frame with 8 averages.

### 2.15 Structured Illumination microscopy

HEK293T cells were grown at a density of 200,000 cells on poly-d-lysine coated 18 × 18 mm 1.5H coverglasses (474030-9000-000; Zeiss, Germany) and cultured for 24 h at 37 °C/5% CO_2_. Cells were then transiently transfected with 3 μg per well of HaloTag VEGFR2 cDNA and grown for 24 h at 37 °C/5% CO_2_. Cells were then incubated with 0.5 μM HaloTag AF488 membrane impermeant ligand (15 min at 37 °C/5% CO_2_; G1002, Promega Corporation, USA) and then washed three times with HBSS/0.1% BSA. For receptor tyrosine kinase inhibitor experiments, cells were pretreated with vehicle or 1 μM cediranib (30 min at 37 °C) and then stimulated with 10 nM VEGF_165_a-TMR (30 min at 37 °C). For timecourse experiments, cells were treated with 3 nM VEGF_165_a-TMR for 5, 30, 60 or 120 min at 37 °C. All cells were then fixed using 3% PFA/PBS (10 min at room temperature), washed with PBS and mounted onto slides using Prolong Gold antifade reagent (P10144; Thermo Fisher Scientific, USA). TetraSpeck™ microspheres (0.1 μm; T7279, Thermo Fisher Scientific, USA) were included in each experiment to allow X/Y/Z channel alignment correction in image processing. Slides were imaged using a Zeiss ELYRA PS.1 microscope using a Plan Apochromat 63×/1.4 oil DIC M27 objective with Zeiss Immersol™ 518F (30 °C) oil (Zeiss, Germany). Multitrack imaging with HaloTag VEGFR2 imaged using bandpass 495–550 plus longpass 750 filter at 1% laser power with 150 ms exposure time (28 μm grating) and VEGF_165_a-TMR with a bandpass 570–620 plus longpass 750 filter at 4% laser power with 50 ms exposure time (34 μm). All images were acquired at 1024 × 1024 frame size over 5 rotations as a Z stack of 30–40 slices. Images were manually processed with consistent raw scaling between and within experiments. (Sectioning of 100 × 83 × 93; Zen Black 2012, Zeiss, Germany).

### 2.16 HUVEC proliferation assay

Human umbilical vein endothelial cells (HUVECs; passage 4–6; C0035C (newborn, single donor) Thermo Fisher Scientific, USA) were grown to confluency in Medium 200 (Thermo Fisher Scientific, USA) supplemented with large vessel endothelial supplement (LVES 50×; Thermo Fisher, USA) at 37 °C/5% CO_2_. Cells were seeded at 5000 cells per 100 μl/per well onto black, clear bottomed 96 well plates (Grenier Bio-One, 655090) and left to grow at 37 °C/5% CO_2_ for 24 h. The next day, the plating medium was replaced with 150 μl per well of Medium 200 with 0.1% serum for a further 24 h at 37 °C/5% CO_2._ On day 3, cells were stimulated in triplicate wells for 24 or 48 h at 37 °C/5% CO_2_ with 3 nM or 30 nM VEGF_165_a (R and D Systems) or VEGF_165_a-TMR in Medium 200/0.1% serum. At the end of the incubation period, cells were washed with PBS (100 μl/well), fixed using 3% PFA/PBS (20 min at room temperature) and nuclei stained using H33342 (2 mg/ml; 15 min at room temperature). Nuclei were imaged at 4 sites per well using a IX Micro widefield platereader (Molecular Devices, USA) fitted with a 4× objective using a DAPI filter with 25 ms exposure time. Nuclei were counted using a granularity algorithm (MetaXpress, Molecular Devices, USA). Data were normalised to show fold increases in proliferation compared to vehicle treatment and expressed as mean ± S.E.M.

### 2.17 Data analysis and statistical tests

Equilibrium binding: All data were analysed using Prism 6 (GraphPad; San Diego, CA, USA). NanoBRET competition experiments were fitted as raw BRET ratios using non-linear least squares regression (variable slope) with no constraints used. Saturation curves were fitted simultaneously for total (VEGF_165_a-TMR alone) and non-specific binding (top concentration of competing VEGF_165_a alone) using both one and two site fits. Background and non-specific binding parameters were shared across all data sets. All saturations fitted preferentially to a one site fit using the equation:

$$\mathrm{Total}\;\mathrm{binding}=\frac{B_{\mathrm{MAX}}x[B]}{[B]+K_{D}}+\mathrm{Mx}[B]+C$$

*where B_max_* is the maximal specific binding, [B] the concentration of fluorescent ligand (nM), *K_D_* the equilibrium dissociation constant (nM), *M* the slope of the non specific binding component and *C* the *y* axis intercept.

Binding affinities (K_i_) of the unlabeled ligands were calculated using the Cheng-Prusoff equation:

$$K_{i}=\frac{{\mathrm{IC}}_{50}}{1+\frac{\left[L\right]}{K_{D}}}$$

where [L] is the concentration of fluorescent ligand used (nM). *K_D_* values (nM) were derived from saturation binding curves. IC_50_ is the molar ligand concentration that will inhibit 50% of the specific binding of the fluorescent ligand concentration [L] and was calculated using the equation:

$$\%\;\mathrm{specific}\;\mathrm{VEGF}\;\mathrm{binding}=\frac{100\times {\mathrm{IC}}_{50}}{[A]+{\mathrm{IC}}_{50}}$$

where [A] is the concentration of competing drug used.

Linear regression analysis was also performed on the relationship between IC_50_ and [VEGF_165_a-TMR] using a variant of the Cheng-Prusoff equation:

$${\mathrm{IC}}_{50}=([\mathrm{VEGF}165a-\mathrm{TMR}]\times K_{i}÷K_{D})+K_{i}$$

*Association kinetics:* Fluorescent ligand binding association kinetic data were fitted to the following mono exponential association function:

$$Y=Y_{\max}\cdot (1-e^{-k_{\mathrm{obs}}\cdot t})$$

Y_max_ equals the level of specific binding at infinite time, t is the time of incubation and k_obs_ is the rate constant for the observed rate of association.

To evaluate whether the data were compatible with a simple mass-action equilibrium interaction with a single non-interaction binding site, the k_obs_ values obtained were compared at different fluorescent ligand concentrations by investigating the linearity of the relationship between them according to the expression:

$$k_{\mathrm{obs}}=k_{\mathrm{on}}\times [L]+k_{\mathrm{off}}$$

where k_off_ is the dissociation rate constant of the ligand in min^–1^ and k_on_ is the association rate constant in M^–1^ min^–1^.

Kinetically determined K_D_ values were calculated from these kinetic parameters using the following equation:

$$K_{D}=\frac{k_{\mathrm{off}}}{k_{\mathrm{on}}}$$

*Concentration response course and time course data obtained from automated imaging:* For VEGF_165_a-TMR concentration response curves of internalization of ligand/VEGFR2 complexes, data were expressed as a percentage of responses obtained using 30 nM VEGF_165_a-TMR (100%) or vehicle (0%). Data were fitted using non-linear least squares regression (variable slope) using GraphPad Prism. All data were expressed as mean ± S.E.M and pooled from 8 independent experiments.

For 120 min time course data of VEGF_165_a-TMR internalization, VEGFR2 activation (measured by anti p1175 or pY1214 labeling) or VEGFR2-Halo internalization, data were normalised as a percentage of peak responses measured at 20 min stimulation (100%) or with no agonist stimulation (0%) respectively. Data for VEGF_165_a-TMR internalization were fitted to a mono exponential association function:

$$Y=Y_{\max}\cdot (1-e^{-k_{\mathrm{obs}}\cdot t})$$

where Y_max_ equals the level of VEGF_165_a-TMR internalization measured at 120 min, t is the time of stimulation and k_obs_ is the rate constant for the observed internalization.

All data were expressed as mean ± S.E.M and pooled from 4/5 independent experiments.

*NFAT reporter genes:* All data obtained from the NFAT luciferase reporter gene assays were normalised to 10 nM VEGF_165_a responses and fitted with non linear regression using the equation as described previously [10].

Statistical analysis used unpaired *t*-test or ANOVA as appropriate (P < 0.05). The n values in the text show the number of separate repeat experiments.

*HUVEC proliferation assays:* All data were expressed as percentage fold increases in cell count following VEGF_165_a or VEGF1_65_a-TMR treatment when normalised to vehicle treatment alone (100%). All data were expressed as mean ± S.E.M. Statistical analysis using one way ANOVA was performed to compare vehicle with ligand treatments (P < 0.001). The n values in the text show the number of separate repeat experiments.

## 3 Results

### 3.1 Synthesis of an active fluorescent variant of the VEGF_165_a homodimer labeled on a single N-terminal cysteine

**Fig. 1:**
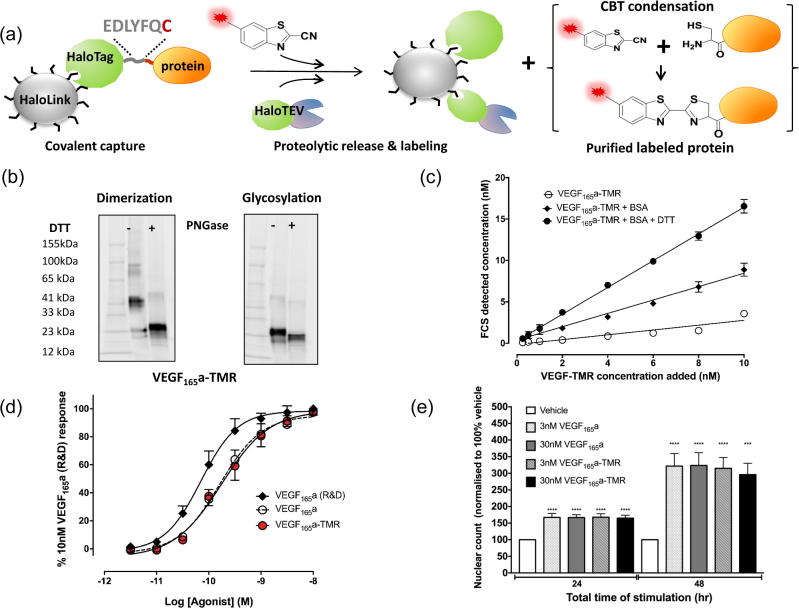
Synthesis and characterisation of purified VEGF_165_a-TMR. (a) Synthetic strategy for purification and labeling of VEGF_165_a-TMR. (b) Fluorescent SDS-PAGE analysis (of VEGF_165_a-TMR (E_ex_ = 532 nm; E_em_ = 580 nm) in the presence or absence of 100 mM DTT and with or without deglycosylation by PNGase. (c) Influence of bovine serum albumin (0.1% BSA) and 10 mM DTT on VEGF_165_a-TMR concentrations measured using fluorescence correlation spectroscopy (FCS). Data are from 3 independent experiments and expressed as mean ± SEM. (d) Stimulation of NFAT luciferase production by HEK293T cells stably expressing VEGFR2 by VEGF_165_a (R&D Systems; closed circles), VEGF_165_a prepared identically to the fluorescent analogue (open circles) or fluorescent VEGF_165_a-TMR (red circles). Values represent mean ± SEM from 4 independent experiments from which quadruplicate determinations were made. Data are expressed as a percentage of the response to 10 nM VEGF_165_a (R&D Systems) obtained in each separate experiment. (e) Effect of VEGF_165_a and VEGFR_165_a-TMR on proliferation of human umbilical endothelial cells (HUVECs). Following stimulation with VEGF_165_a or VEGF_165_a-TMR (3 or 30 nM) for 24 or 48 h, HUVECs were fixed using 3% PFA/PBS and the nuclei stained using H33342 (2 mg/ml). Cells were imaged using a IX Micro widefield platereader at 4× magnification and nuclei were counted using a granularity algorithm (MetaXpress, Molecular Devices). Data are presented as fold increases in proliferation compared to vehicle treatment (mean ± S.E.M) and are pooled from 5 individual experiments. One way ANOVA was used to determine the statistical significance of ligand treatment when compared to vehicle only for both 24 and 48 h treatments (^***^P < 0.0002; ^****^P < 0.0001). (For interpretation of the references to colour in this figure legend, the reader is referred to the web version of this article.)

Synthesis and purification of a fluorescent variant of VEGF_165_a labeled on a single amino acid was achieved as depicted in Fig.1a. Briefly, VEGF_165_a was genetically fused to an N terminal HaloTag via a short amino acid linker containing a modified TEV recognition site (EDLYFQC), which upon proteolytic cleavage releases VEGF_165_a with an N terminal cysteine residue (cys-VEGF_165_a). The secreted fusion protein expressed in HEK293T cells was covalently captured onto HaloLink beads [33]. Cys-VEGF_165_a was released from the beads by proteolytic cleavage using HaloTEV protease, while HaloTag and HaloTEV remained permanently attached to the beads eliminating the need for post cleavage removal. Proteolytic cleavage in the presence of TMR fluorophore coupled to 2-cyanobenzothiazole (6-TMR-PEG-CBT) [31] enabled site specific labeling of the released cys-VEGF_165_a via condensation of 6-TMR-PEG-CBT and the exposed N terminal cysteine [34]. This purification and labeling reaction were performed in a physiological buffer under reducing conditions (100 µM *tris*(2-carboxyethyl)phosphine; TCEP). The purified and labeled VEGF_165_a (VEGF_165_a-TMR) was collected and dialyzed to allow final formation of the di-sulphide linked anti-parallel VEGF_165_a homodimer under non-reducing conditions.

**Fig. 2:**
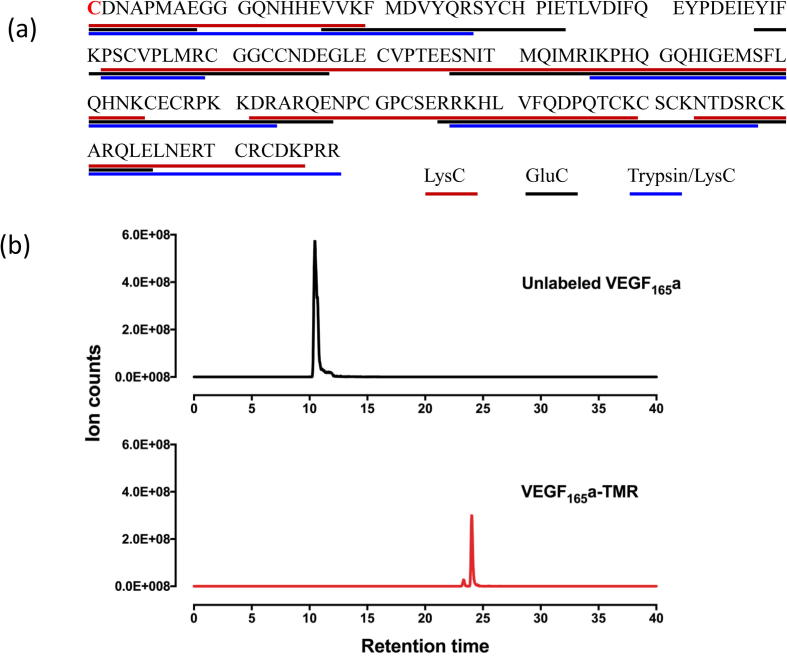
LC-MS/MS analysis of VEGF_165_a-TMR digested with multiple proteases. (a) Peptide coverage achieved by digestion with LysC, GluC and Trypsin/LysC proteases. The N terminal cysteine is marked in red. None of the other 16 residues presented in the VEGF_165_a protomer were labeled. Protein identity was confirmed by searching the MS/MS spectra using the Mascot search engine (Matrix Science Inc, Boston, USA) against a human database (SwissProt). The highest scoring hit was the VEGF sequence. (b) LC-MS/MS analysis of the peptide containing the N terminal cysteine (CDNAPMAEGGGQNHHEVVK) derived from VEGF_165_a and VEGF_165_a- that were purified in the same manner and digested with LysC protease. Retention times and mono-isotopic masses are given in Table 1. (For interpretation of the references to colour in this figure legend, the reader is referred to the web version of this article.)

Fluorescent SDS-PAGE analysis of the purified VEGF_165_a-TMR in the presence or absence of 100 mM dithiothreitol (DTT) confirmed that, in non-reducing conditions, VEGF_165_a-TMR was largely present as a homodimer (Fig.1b). Deglycosylation by PNGase provided evidence that the purified VEGF_165_a-TMR was glycosylated (Fig.1b). Labeling efficiency and specificity of the VEGF_165_a protomer with 6-TMR-PEG-CBT was determined by liquid chromatography-tandem mass spectrometry (LC-MS/MS) analysis of labeled and unlabeled VEGF_165_a digested with multiple proteases. This analysis indicated highly efficient and selective labeling of the N terminal cysteine residue (Fig. 2 and Table 1). 6-TMR-PEG-CBT modification was identified exclusively on the N-terminal cysteine residue at >98% labeling efficiency. We did not observe non-specific labeling of any of the other 16 cysteine residues present in the VEGF_165_a protomer.

Using an NFAT reporter gene assay [10], we compared the agonist activity of VEGF_165_a-TMR to non-fluorescent VEGF_165_a (purified in an identical manner to VEGF_165_a-TMR) and VEGF_165_a obtained from a commercial source (R&D systems) in cells expressing the wild-type VEGFR2 receptor (pEC_50_ values were 9.78 ± 0.07, 9.72 ± 0.09 and 10.14 ± 0.07; n = 4 in each case). Our results indicated that the two VEGF_165_a proteins exhibited small differences in their potencies that may be related to the small modification in the N-terminus depicted in Fig.1a. Comparison of the potencies of VEGF_165_a and VEGF_165_a-TMR purified in the same manner, however, indicated that they had identical potencies (Fig.1d). VEGF_165_a and VEGF_165_a-TMR also were able to stimulate the proliferation of HUVECs to a similar extent (Fig.1e).

### 3.2 VEGF_165_a-TMR exists as a homodimer in physiological solutions

To verify that VEGF_165_a-TMR existed as a homodimer in physiological solutions, we undertook fluorescence correlation spectroscopy (FCS) analysis of the fluorescent ligand in solution (Fig.1c). This is a single molecule approach that allows the number of fluorescence particles (and hence concentration) and their diffusion coefficients to be measured [32], [35]. Experiments performed in Hank’s buffered saline solution (HBSS) in the presence of 0.1% protease-free bovine serum albumin (BSA) maintained a constant concentration of VEGF_165_a-TMR in solution for at least 1 h (data not shown). The final concentrations obtained in solution were very similar to the concentrations calculated for an antiparallel dimer (Fig.1c). The slope of the linear regression in Fig.1c in the presence of 0.1% BSA was 0.81 (R^2^ = 0.87; 95% confidence limits 0.71–0.90). In the absence of BSA, however, the free concentrations of VEGF_165_a-TMR were markedly reduced (slope = 0.20; R^2^ = 0.84; 95% confidence limits 0.25–0.33; Fig.1c). In the presence of 10 mM DTT, there was a twofold increase in the number of fluorescence particles at each concentration (slope = 1.62; R^2^ = 0.98; 95% confidence limits 1.54–1.69; Fig.1c). These data are consistent with the presence of an antiparallel VEGF_165_a-TMR dimer in physiological solution, labeled on each protomer with a single fluorophore in non-reducing conditions.

### 3.3 Evaluation of the specific binding of VEGF_165_a-TMR to NanoLuc-tagged VEGFR2

**Fig. 3:**
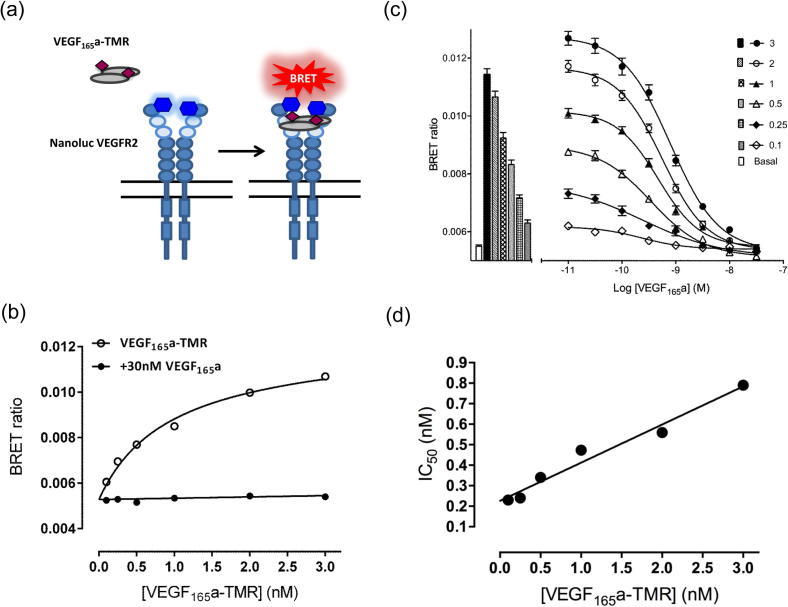
Binding characteristics of VEGF_165_a-TMR to VEGFR2 in HEK293T cells. (a) Schematic of the use of NanoBRET to investigate the binding of VEGF_165_a-TMR to an N terminal NanoLuc tagged VEGFR2 (NLuc-VEGFR2). (b) Saturation binding of increasing concentrations of VEGF_165_a-TMR in the presence or absence of unlabeled VEGF_165_a (30 nM; to define non-specific binding). Data are expressed as raw BRET ratios. VEGF_165_a-TMR (0.1–3 nM) and unlabeled VEGF_165_a (30 nM) were added simultaneously to triplicate wells and incubated for 60 min at 37 °C. Values represent mean ± S.E.M of five independent experiments performed independently of displacement experiments shown in (c). Where not shown, the error bars are within the size of the symbol. (c) Inhibition of the binding of VEGF_165_a-TMR (0.1–3 nM) to NanoLuc-tagged VEGFR2 by increasing concentrations of unlabeled VEGF_165_a. VEGF_165_a-TMR (0.1–3 nM) and unlabeled VEGF_165_a were added simultaneously and incubated for 60 min at 37 °C. Values represent mean ± S.E.M of five independent experiments. Bars represent the binding of VEGF_165_a-TMR obtained at each fluorescent ligand concentration in the absence of competing unlabeled VEGF_165_a. The open bar shows the BRET ratio obtained in the absence of either fluorescent or unlabeled ligand. (d) Linear regression analysis (R^2^ = 0.97; p < 0.001) of the relationship between IC_50_ value determined in (c) and the concentration of VEGF_165_a-TMR. The y intercept provides an estimate for the K_i_ of competing VEGF_165_a (0.22 nM) and the slope (0.19) represents the ratio K_i_/K_D_ yielding a value of 0.85 nM for the K_D_ of VEGF_165_a-TMR.

The use of BRET and a new bright luciferase (NanoLuc) provided the means to monitor ligand-binding interactions between VEGF_165_a-TMR and full length VEGFR2 in living cells, and in real time. Human VEGFR2 was labeled on its N-terminus with NanoLuc and expressed in HEK293T cells (NLuc-VEGFR2). A schematic of the proposed interaction between an antiparallel VEGF_165_a-TMR and Ig-like domains 2 and 3 of a VEGFR2 homodimer is shown in Fig.3a. Saturable binding of VEGF_165_a-TMR detected by BRET (K_D_ = 0.92 ± 0.10 nM; n = 5; 60 min incubation) was largely prevented by 30 nM VEGF_165_a (Fig.3b). At each concentration of VEGF_165_a-TMR (0.1–3 nM), competitive inhibition of receptor-specific binding could be achieved in the presence of increasing concentrations of VEGF_165_a, yielding an apparent pK_i_ of 9.81 ± 0.14 (assuming mass action kinetics; n = 5; Table 2; Fig.3c). Analysis of the linear relationship (R^2^ = 0.97; p < 0.001) between IC_50_ and the concentration of VEGF_165_a-TMR, assuming a competitive interaction, indicated that the K_i_ value of VEGF_165_a was 0.22 nM and the K_D_ (for VEGF_165_a-TMR) was 0.85 nM (Fig.3d).

**Table 2:** Summary of the binding properties and functional efficacies of VEGF ligand splice variants in HEK293T NLuc VEGFR2 cells. Data are mean ± S.E.M of 5 separate experiments. Each individual experiment was performed in triplicate. In the NFAT reporter gene assays 10 nM VEGF_165_a was included in each experiment and maximal responses are expressed relative to this. For the NanoBRET assays IC_50_ values were converted to pK_i_ values using the Cheng-Prusoff equation.

Ligand	NanoBRET	NFAT Luciferase reporter gene assay	
	pK_i_	pEC_50_	% Maximum response (VEGF_165_a = 100%)
VEGF_165_a	9.81 ± 0.14	9.90 ± 0.06	100
VEGF_165_b	9.41 ± 0.12	9.38 ± 0.06	68.1 ± 5.7
VEGF_189_a	8.99 ± 0.09	9.51 ± 0.11	71.1 ± 3.6
VEGF_145_a	8.74 ± 0.14	9.25 ± 0.09	71.6 ± 3.9
VEGF_121_a	9.47 ± 0.07	9.13 ± 0.20	65.9 ± 8.8

### 3.4 Inhibition of VEGF_165_a-TMR binding by VEGF-A isoforms

**Fig. 4:**
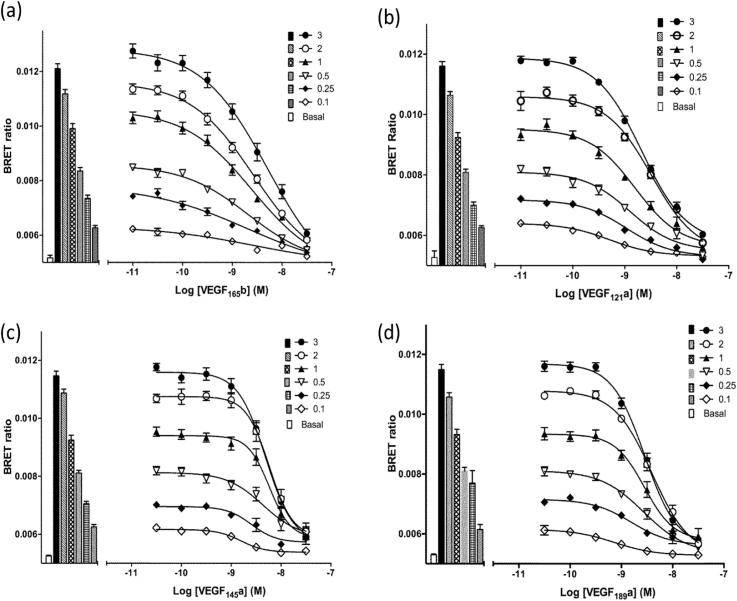
Inhibition of the binding of VEGF_165_a-TMR to NLuc-VEGFR2 by unlabeled VEGF isoforms. Fixed concentrations of VEGF_165_a-TMR (0.1–3 nM) were added simultaneously with increasing concentrations of (a) VEGF_165_b, (b) VEGF_121_a, (c) VEGF_145_a and (d) VEGF_189_a, and incubated for 1 h at 37 °C. Data represent mean ± S.E.M of 5 independent experiments, each performed in triplicate. Bars represent the binding of VEGF_165_a-TMR obtained at each fluorescent ligand concentration in the absence of competing ligand. The open bar shows the BRET ratio obtained in the absence of VEGF_165_a-TMR.

In addition to VEGF_165_a, a number of other isoforms of VEGF-A were investigated as inhibitors of VEGF_165_a-TMR binding to VEGFR2 (Fig. 4). VEGF_189_a, VEGF_165_b, VEGF_145_a and VEGF_121_a were all potent inhibitors of binding (Fig. 4; Table 2). The pK_i_ values obtained for each isoform reconciled with the pEC_50_ values obtained in a functional NFAT reporter gene assay in the same cell line (Table 2).

### 3.5 Association kinetics of VEGF_165_a-TMR binding to NLuc-VEGFR2

**Fig. 5:**
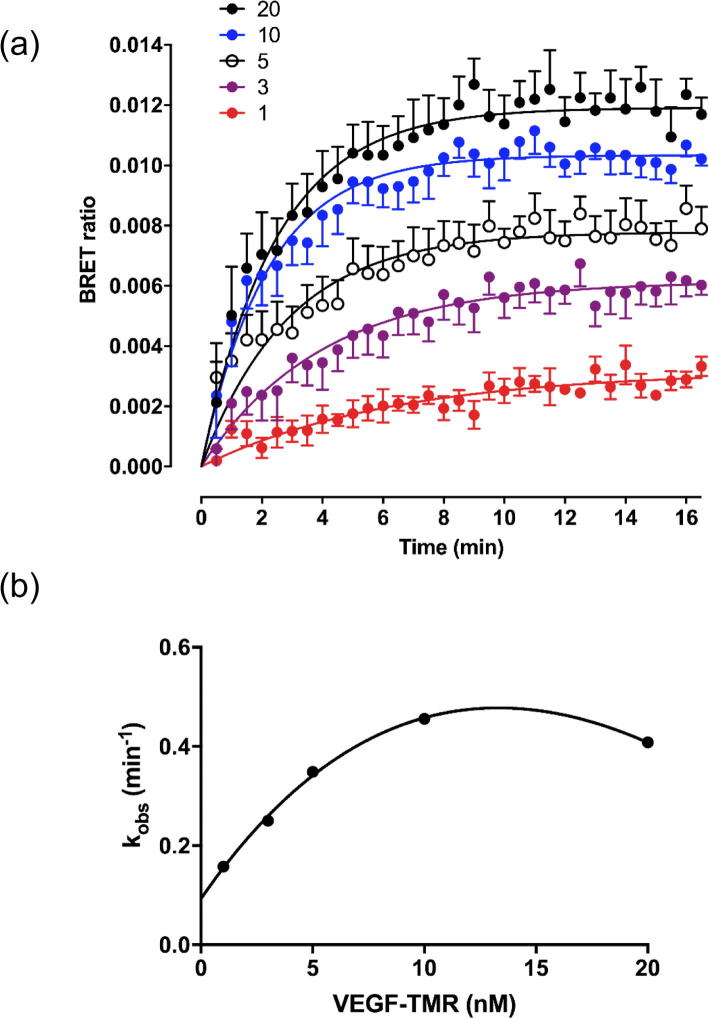
Ligand binding kinetics of VEGF_165_a-TMR to VEGFR2. (a) Time course of VEGF_165_a-TMR binding to NLuc-VEGFR2. HEK293T cells expressing NLuc-VEGFR2 were treated with furimazine and the plate left to equilibrate for 5 min. Vehicle or VEGF_165_a-TMR (1–20 nM; coloured symbols) was then added to the appropriate wells, and measurements made every 30 s for 16 min at 37 °C. Data represent mean ± S.E.M from 5 independent experiments. Individual curves were fitted to a simple exponential association model to obtain K_obs_. (b) A plot of K_obs_ values obtained in (a) with increasing concentrations of VEGF_165_a-TMR.

The ability to directly monitor in real time the binding of VEGF_165_a-TMR to NLuc-VEGFR2 in living cells provided an opportunity to evaluate the kinetic parameters of ligand binding (Fig.5a). Kinetics analysis was undertaken for five different concentrations of VEGF_165_a-TMR (1–20 nM) and the data were fitted to a simple association exponential model (Fig.5a). An evaluation of the k_obs_ value obtained at each fluorescent ligand concentration indicated that the linear relationship expected for a simple mass-action equilibrium was not observed (Fig.5b), and k_obs_ was lower than expected at high ligand concentrations. At the three lowest concentrations of VEGF_165_a-TMR used, there was a linear relationship that allowed estimation of k_on_ (4.78 × 10^7^ min^−1^ M^−1^) and k_off_ (0.11 min^−1^) yielding a kinetic K_D_ of 2.27 nM.

### 3.6 The effect of receptor tyrosine kinase inhibitors on the binding of VEGF_165_a-TMR to VEGFR2

**Fig. 6:**
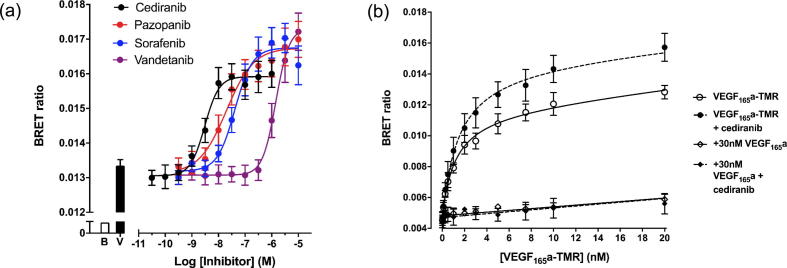
Effect of receptor tyrosine kinase inhibitors (RTKIs) on VEGFR2 ligand binding. (a) NLuc VEGFR2 cells were pre-treated for 30 min at 37 °C with cediranib, pazopanib, sorafenib or vandetanib prior to the addition of 10 nM VEGF_165_a-TMR for 60 min at 37 °C. The bars represent cells stimulated with vehicle (B; open bar; basal) or 10 nM VEGF_165_a-TMR in the absence of inhibitor (V; filled bar). Data represent mean ± S.E.M from 4 independent experiments in which triplicate determinations were made. (b) VEGF_165_a-TMR binding to NLuc-VEGFR2 in the absence (solid lines) or presence (dotted lines; 30 min pre-incubation) of 1 μM cediranib. Data are mean ± S.E.M of four independent experiments.

Receptor tyrosine kinases inhibitors (RTKIs) targeted at VEGFR2 have proved to be attractive approaches to cancer therapy [3]. Here we have investigated whether they have an allosteric effect on the binding of VEGF to its receptor. Initial studies evaluated the impact of four RTKIs on the specific binding of 10 nM VEGF_165_a-TMR to NLuc-VEGFR2 using BRET following 60 min incubation with VEGF_165_a (Fig.6a). Cediranib, pazopanib, sorafenib and vandetanib all caused a concentration-dependent increase in the binding of 10 nM VEGF_165_a-TMR (maximal responses were 139.0 ± 4.4, 144.8 ± 3.5, 149.3 ± 3.2 and 152.8 ± 7.1% respectively, relative to binding of VEGF_165_a-TMR alone; n = 4; Fig.6a; Table 3). The EC_50_ values obtained for each RTKI for this increase in binding were similar to the IC_50_ values obtained for these compounds from inhibition of the NFAT reporter gene response to 1 nM VEGF_165_a in the same cells (Table 3). Analysis of the saturation binding curves for VEGF_165_a-TMR binding to NLuc-VEGFR2, in the presence and absence of 1 μM cediranib, (Fig.6b) confirmed that this effect was on B_MAX_ and did not change the K_D_ value of VEGF_165_a-TMR (K_D_ = 1.18 ± 0.56 nM and 1.14 ± 0.44 nM in the presence and absence of 1 μM cediranib).

**Table 3:** Summary of the effect of RTKIs on 10 nM VEGF_165_a-TMR induced BRET ratios in HEK293T NLuc VEGFR2 cells. For NanoBRET binding assays cells were pretreated with a RTKI for 30 min prior to addition of 10 nM VEGF_165_a-TMR for a further 60 min. Data are expressed as mean ± SEM and the number of separate experiments is given in parentheses. Each individual experiment was performed in triplicate. For NFAT reporter gene assays, cells were treated with each RTKI for 60 min prior to the addition of 1 nM VEGF_165_a for 5 h.

Inhibitor	NanoBRET		NFAT Luciferase
	pEC_50_	% Increase in binding of 10 nM VEGF_165_a-TMR	pIC_50_
Cediranib	8.52 ± 0.13 (4)	139.0 ± 2.0 (4)	9.78 ± 0.73 (6)
Pazopanib	7.70 ± 0.24 (4)	144.8 ± 3.5 (4)	8.55 ± 0.19 (5)
Sorafenib	7.38 ± 0.14 (4)	149.3 ± 3.2 (4)	7.77 ± 0.45 (6)
Vandetanib	5.84 ± 0.12 (4)	152.8 ± 7.1 (4)	6.26 ± 0.27 (5)

**Fig. 7:**
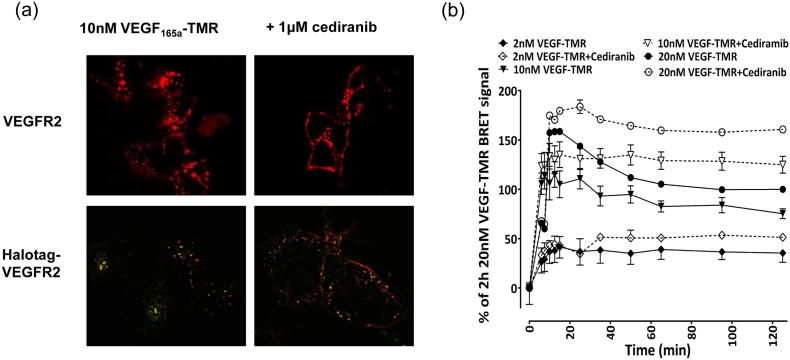
Cellular location and time course of VEGF_165_a-TMR binding to NLuc-VEGFR2. (a) Confocal (upper panels) (Zeiss LSM 510) or (low panels) Structured Illumination Microscopy (SIM) super resolution images of cells pre-treated with vehicle or 1 μM cediranib for 30 min at 37 °C followed by stimulation with 10 nM VEGF_165_a-TMR for a further 30 min at 37 °C. Images are representative of 3 independent experiments. (b) Influence of incubation time on NanoBRET ligand-binding characteristics. Cells were pre-treated with vehicle (solid lines) or 1 μM cediranib (dotted lines) for 30 min at 37 °C, prior to stimulation with 2 nM, 10 nM or 20 nM VEGF_165_a-TMR over 120 min at 37 °C. Data (mean ± S.E.M) are expressed as a percentage of the BRET ratio obtained at 120 min with 20 nM VEGF_165_a-TMR from four independent experiments.

**Fig. 8:**
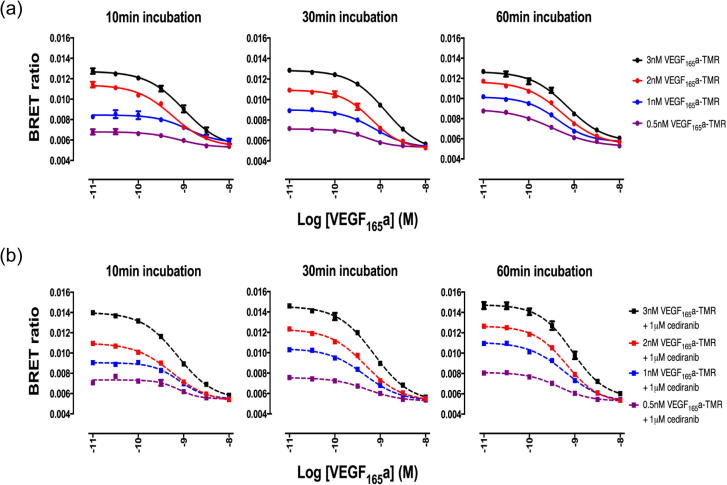
Influence of incubation time on ligand binding competition experiments in the presence or absence of cediranib. HEK293T NLuc VEGFR2 cells were pretreated with either (a) vehicle or (b) 1 μM cediranib for 30 min at 37 °C prior to continued incubation in the presence of VEGF_165_a-TMR (0.5–3 nM) and various concentrations of VEGF_165_a for 10, 30 or 60 min at 37 °C. Data represent mean ± S.E.M from 3 to 4 independent experiments.

The availability of a red fluorescently tagged variant of VEGF_165_a also provided an opportunity to evaluate the cellular location of VEGF_165_a-TMR in living cells using fluorescence microscopy (Fig.7a). In the absence of RTKIs, VEGF_165_a-TMR (applied at 10 nM) was largely visible in intracellular vesicles after 30 min incubation (Fig.7a). However, it was striking that after a 30 min pre-incubation period with cediranib, bright VEGF_165_a-TMR fluorescence was largely localised to the cell membrane (Fig.7a). Structured illumination super resolution microscopy (SIM) to evaluate the co-localisation of HaloTag-VEGFR2 and VEGF_165_a-TMR binding confirmed these observations (Fig.7a). The time course of the effect of cediranib on the binding of 2, 10 and 20 nM VEGF_165_a-TMR is shown in Fig.7b. Over incubation times > 20 min, there was a reduction in the BRET signal that can be prevented by cediranib (Fig.7b). However, the ability of VEGF_165_a to compete with VEGF_165_a-TMR binding was very similar at all time points studied (Fig. 8) and yielded similar pK_i_ values (Table 4).

**Table 4:** Summary of NanoBRET derived binding affinity measurements when VEGF_165_a-TMR and VEGF_165_a are simultaneously co-incubated for set incubation times in the presence or absence of cediranib. Values are mean ± SEM of 3–4 separate experiments. pK_i_ values determined from individual experiments summarised in Fig. 8.

Ligand incubation time (min)	VEGF_165_a pK_i_	
		Plus 1 μM cediranib
10	8.93 ± 0.09	9.17 ± 0.04
30	8.99 ± 0.10	9.23 ± 0.06
60	9.28 ± 0.06	9.18 ± 0.06

### 3.7 Impact of VEGFR2 internalization and ligand dissociation within intracellular endosomes

**Fig. 9:**
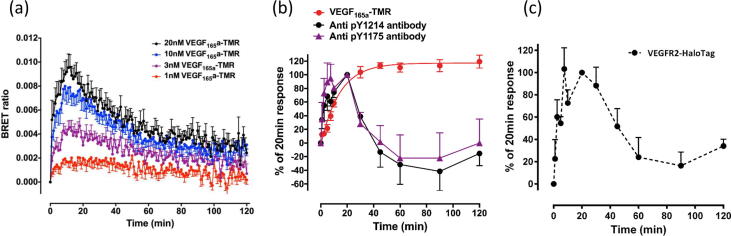
Internalization and activation status of VEGFR2 in response to VEGF_165_a. (a) Time course of VEGF_165_a-TMR binding to NLuc-VEGFR2. Furimazine was added 5 mins prior to VEGF_165_a-TMR. (b) Time course of the internalization of VEGF_165_a-TMR (red circles) and activated VEGFR2 (stained with antibodies targeting phosphate moieties indicative of VEGFR2 activation) within the same cells. 3 nM VEGF_165_a-TMR was added at set time points (1–120 min). Cells were fixed using 3% PFA/PBS, permeabilised (0.025% Triton-X-100/PBS) and labeled with a rabbit antibody targeting pY1175 (purple triangles) or pY1214 of VEGFR2 (black circles). Cells were imaged using a IX Micro widefield platereader. Data are expressed as a percentage of the peak responses at 20 min and represent mean ± S.E.M from 3 independent experiments. (c) Timecourse of 3 nM VEGF_165_a induced internalization of VEGFR2-HaloTag. Cells were labeled with 0.5 μM HaloTag-TMR fluorescent ligand as described under Methods. Images were analysed and data expressed as described for (b). (For interpretation of the references to colour in this figure legend, the reader is referred to the web version of this article.)

**Fig. 10:**
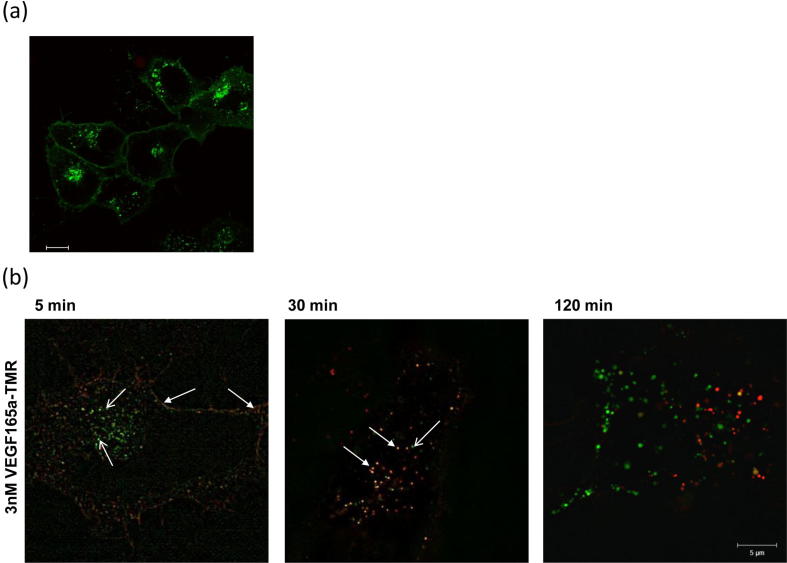
(a) Confocal imaging in HEK293T cells transiently expressing HaloTag VEGFR2. HEK293T cells stably expressing HaloTag VEGFR2 were treated with vehicle for 30 min at 37 °C. All cells were imaged live using a Zeiss LSM Exciter and are representative images from 3 independent experiments. (b). Structured Illumination Microscopy (SIM) super resolution images of HEK293T cells transiently transfected with HaloTag VEGFR2 (green) and treated for distinct time points with 3 nM VEGF_165_a (red; 5, 30 or 120 min at 37 °C). Areas of colocalisation are shown in yellow and examples shown by the closed white arrow. Examples of non-localised intracellular HaloTag VEGFR2 are illustrated by the open white arrow.

The real-time kinetics of ligand-binding to NLuc VEGFR2 over longer incubation times are shown in Fig.9a. Up to 2 h after addition of VEGF_165_a-TMR there was still a measurable BRET signal at all concentrations of the fluorescent ligand indicating that some of the VEGF_165_a-TMR and NLuc-tagged receptor are still in very close proximity at these later incubation times (Fig.9a). The ability of RTKIs to inhibit both VEGFR2 internalization induced by high concentrations of VEGF_165_a-TMR (Fig.8a) and the reduction in the NanoBRET signal (Fig.8b) raises two possibilities: (1) the reduction in BRET signal over long incubation times in the absence of RTKIs is a consequence of the different local environment of intracellular endosomes or (2) it is due to VEGF_165_a-TMR/VEGFR2 dissociation within the acidic and proteolytic environment of the intracellular endosomes. Some insights into the mechanisms underlying these observations are provided by a comparison of the temporal profile of the NanoBRET signal (Fig.9a) and the extent of the appearance of phosphorylated active VEGFR2 (Y1214 or Y1175) and VEGF_165_a-TMR in intracellular vesicles following addition of fluorescent VEGF_165_a-TMR (Fig.9b). The profile of pY1214 or pY1175-VEGFR2 within intracellular vesicles essentially follows that of the NanoBRET signal (Fig.9b). In contrast, the appearance of VEGF_165_a-TMR within intracellular vesicles lags the phosphorylation of VEGFR2 and is sustained for 2 h (Fig.9b). The internalization (t_1/2_ = 9.18 ± 0.71 min; 95% confidence limits = 6.93–11.41; n = 4) of VEGF_165_a-TMR was concentration dependent (pEC_50_ = 8.68 ± 0.03; n = 8) and inhibited by co-incubation with VEGF_165_a (pIC_50_ 9.68 ± 0.10, n = 6; for inhibition of the internalization of 3 nM VEGF_165_a-TMR). To confirm the temporal profile of total VEGFR2 internalization following stimulation with VEGF_165_a, we also generated a stable cell line expressing VEGFR2 with a HaloTag on its C-terminus. Stimulation of these cells with 3 nM VEGF_165_a produced a rapid internalization of VEGFR2 into intracellular vesicles that peaked between 10 and 20 min (Fig.9c) and then followed the same time course as the phosphorylated receptor (Fig.9b) and the VEGF_165_a-TMR ligand binding BRET signal (Fig.9a). Taken together, these data suggest that VEGFR2 is internalized by VEGF_165_a-TMR into intracellular endosomes where the BRET signal is decreased over time as a consequence of the dissociation of the fluorescent ligand from the internalized receptor. Confocal microscopy of N-terminal HaloTag VEGFR2 under basal conditions showed that a significant proportion was constitutively internalized, with the remainder on the cell membrane (Fig.10a). Super-resolution SIM of the co-localisation of VEGF_165_a-TMR and N-terminal HaloTag VEGFR2 showed that it was confined to the cell membrane at early time points (5 min) after addition of the fluorescent agonist and fully colocalised in intracellular vesicles after 30 min (Fig.10b). However, following longer incubation times (120 min) this co-localisation is lost and free HaloTag VEGFR2 returned to the cell surface (Fig.10b).

## 4 Discussion

This study has provided for the first time quantitative information on the real time binding of VEGF-A isoforms to full length VEGFR2 in living cells. Analysis of the specific binding of VEGF_165_a-TMR to NLuc-VEGFR2 indicated an apparent K_D_ value of 0.92 nM that was very similar to the EC_50_ value obtained with this labeled VEGF_165_a analogue in NFAT reporter gene assays. Furthermore, competition experiments also enabled the apparent pK_i_ values for a range of VEGF-A isoforms to be determined. These values were in close agreement with the pEC_50_ values obtained in a functional NFAT reporter gene assay in the same cells. Furthermore, the pK_i_ values for VEGF_165_a and VEGF_165_b were also similar to those determined with ^125^I-labeled VEGF_165_a [5], [7] or by the operational model of Black & Leff from functional NFAT responses [10].

Kinetic analysis of the binding of the antiparallel dimeric VEGF_165_a-TMR to NLuc-VEGFR2 over the first 16 min (before significant receptor internalization occurred) indicated that the data fitted well to a simple association exponential. However, the k_obs_ values obtained with each concentration of fluorescent VEGF_165_a from this analysis did not fit with the linear relationship expected for a simple mass-action equilibrium with non-interacting binding sites. However, at the three lowest concentrations of VEGF_165_a-TMR used, there was a linear relationship that allowed a rough estimation of k_on_ (4.78 × 10^7^ min^−1^ M^−1^) and k_off_ (0.11 min^−1^) to be made yielding a kinetic K_D_ (2.3 nM) that was very similar to the value obtained from equilibrium determinations above.

Receptor tyrosine kinases inhibitors (RTKIs) targeted at VEGFR2 have proved to be attractive approaches to cancer therapy based on their ability to reduce angiogenesis and/or lymphangiogenesis [3]. Here we used the NanoBRET assay to evaluate whether RTKIs had an allosteric effect on the direct binding of VEGF_165_a-TMR to NLuc-VEGFR2 in living cells. Surprisingly, all four compounds studied increased the maximum binding (measured at 60 min) of VEGF_165_a-TMR by 40–50% without altering its K_D_. However, a noticeable feature of their action was that they largely prevented the internalization of VEGFR2 induced by high concentrations of VEGF_165_a-TMR. In the absence of RTKIs, the binding of 10 nM VEGF_165_a-TMR appeared to be largely intracellular after 30 min incubation.

This agonist-induced receptor internalization was time and concentration dependent, and could be monitored in a quantitative manner using high content imaging of the location of VEGF_165_a-TMR, phosphorylated VEGFR2 (pY1214 and pY1175) or VEGFR2 attached to a C terminal HaloTag. It was noticeable that there was a sustained BRET signal (between NLuc-VEGFR2 and VEGF_165_a-TMR) that lasted for up to 2 h, suggesting that some of the fluorescent ligand and NLuc-VEGFR2 remain in close proximity within endosomes [36]. There was, however, a noticeable decrease in this BRET signal over longer incubation times at high concentrations of VEGF_165_a-TMR, which could reflect the influence of the local acidic environment of intracellular endosomes. However, the differential time courses of the ligand-binding BRET signal, internalized active phosphorylated VEGFR2 and the appearance of VEGF_165_a-TMR in intracellular endosomes points to a different conclusion. The similarity of the time courses for NanoBRET, pY1214 or pT1175 VEGFR2 and VEGFR2-HaloTag internalization suggests that this reflects the appearance of agonist-occupied ‘active’ VEGFR2 in intracellular endosomes. This taken alongside the highly localised intracellular VEGF_165_a-TMR fluorescence at later time points suggests that the ligand has dissociated from the receptor in the acidic environment of the intracellular endosomes and allowed VEGFR2 to recycle back to the plasma membrane, consistent with previous observations of the rapid constitutive recycling of VEGFR2 [37], or be degraded within lysosomes [38]. This conclusion is supported by SIM analysis of the subcellular co-localisation of HaloTag-VEGFR2 and VEGF_165_a-TMR at different time points.

In summary, we have used novel protein chemistry to generate a fluorescent variant of VEGF (VEGF_165_a-TMR) labeled on a single cysteine within each protomer of the antiparallel VEGF homodimer. This has been used in conjunction with full length VEGFR2, tagged with the bioluminescent protein NanoLuc, to undertake a real time quantitative evaluation of VEGFR2 and binding characteristics in living cells using bioluminescence energy transfer (BRET). At longer incubation times, VEGFR2 is internalized by VEGF_165_a-TMR into intracellular endosomes where the BRET signal is decreased over time as a consequence of the dissociation of agonist from the receptor and receptor recycling. This internalization can be prevented by the receptor tyrosine kinase inhibitors (RTKIs) cediranib, sorafenib, pazopanib or vandetanib. This new understanding of VEGFR2 ligand-receptor interactions should provide new insights and opportunities for future drug discovery efforts aimed at cancer angiogenesis.

## Author contributions

Conceived the study: Hill, Woolard.

Generated reagents: Friedman Ohana, Zimmerman.

Participated in research design: Kilpatrick, Robers, Hill, Woolard.

Conducted experiments: Kilpatrick, Friedman Ohana, Riching, Machleidt, Wheal, Alcobia, Peach.

Performed data analysis: Kilpatrick, Alcobia, Briddon, Friedman Ohana, Hill.

Wrote or contributed to the writing of the manuscript: Kilpatrick, Wood, Robers, Friedman Ohana, Woolard, Hill.

## Conflict of interest

RFO, KR, MBR, KZ, TM and KVW are employees of Promega Corporation, which has proprietary rights over the NanoBRET assay, HaloTag technology and VEGF_165_a-TMR.
